# Justice, Transparency and the Guiding Principles of the UK’s National Institute for Health and Care Excellence

**DOI:** 10.1007/s10728-021-00444-y

**Published:** 2021-11-08

**Authors:** Victoria Charlton

**Affiliations:** Department of Global Health and Social Medicine, King’s College London, 40 Aldwych, London, WC2B 4BG United Kingdom

**Keywords:** Health technology assessment, Healthcare priority-setting, National Institute for Health and Care Excellence, Procedural justice, Transparency, Value judgements

## Abstract

The National Institute for Health and Care Excellence (NICE) is the UK’s primary healthcare priority-setting body, responsible for advising the National Health Service in England on which technologies to fund and which to reject. Until recently, the normative approach underlying this advice was described in a 2008 document entitled ‘Social value judgements: Principles for the development of NICE guidance’ (SVJ). In January 2020, however, NICE replaced SVJ with a new articulation of its guiding principles. Given the significant evolution of NICE’s methods between 2008 and 2020, this study examines whether this new document (‘Principles’) offers a transparent account of NICE’s current normative approach. It finds that it does not, deriving much of its content directly from SVJ and failing to fully acknowledge or explain how and why NICE’s approach has since changed. In particular, Principles is found to offer a largely procedural account of NICE decision-making, despite evidence of the increasing reliance of NICE’s methods on substantive decision-rules and ‘modifiers’ that cannot be justified in purely procedural terms. Thus, while Principles tells NICE’s stakeholders much about how the organisation goes about the process of decision-making, it tells them little about the substantive grounds on which its decisions are now based. It is therefore argued that Principles does not offer a transparent account of NICE’s normative approach (either alone, or alongside other documents) and that, given NICE’s reliance on transparency as a requirement of procedural justice, NICE does not in this respect satisfy its own specification of a just decision-maker.

## Introduction

In most healthcare systems, the availability of potentially beneficial interventions surpasses the available resources, necessitating decisions about which interventions to adopt and which to reject [1, 10, 41, 43]. In the UK, these decisions are frequently informed by the National Institute for Health and Care Excellence (NICE), whose advice plays a major role in determining which healthcare technologies users of the National Health Service (NHS) in England can access [34]. NICE’s advice inevitably rests on value judgements about how the needs of different NHS users should be balanced and prioritised. Given that there is no societal consensus on such matters, NICE has historically sought to ensure that its decisions can be accepted as legitimate and fair by grounding its approach on two normative frameworks: one that sets out the requirements for a just procedure (based on ‘accountability for reasonableness’, AfR) and another that sets out NICE’s general substantive reasons for deciding which technologies to recommend (based on an ‘ethics of opportunity costs’) [35, 36, 39, 40]. (Table 1).

**Table 1:** NICE’s general normative approach (as set out in SVJ)

SVJ was first published by NICE in 2005 and was later revised and updated in 2008 [35, 36]. The purpose of the document was to describe “the principles NICE should follow when applying social value judgements to the processes it uses to develop guidance as well as during the development of individual forms of guidance” [36]
The principles set out in SVJ are organised around two main ethical frameworks:
*Accountability for reasonableness (AfR)* is a procedural framework for healthcare priority-setting that was developed by Norman Daniels and James Sabin in the 1990s [7]. It rests on the assumption that “in pluralist societies we are likely to find reasonable disagreement about principles that should govern priority setting” and that, “in the absence of consensus on principles, a fair process allows us to agree on what is legitimate and fair” [6]. AfR defines a fair process as one that fulfils four conditions: (1) that both the decisions made and the grounds for reaching them are made public (‘publicity’); (2) that these grounds are ones that fair-minded people would agree are relevant in the particular context (‘relevance’); (3) that there are opportunities for challenging and revising decisions and resolving disputes (‘appeal and revision’), and (4) that measures are in place to ensure that the first three conditions are met (‘enforcement’) [7]. NICE explicitly subscribes to this framework in SVJ, stating that “procedural justice provides for ‘accountability for reasonableness’” and that the procedural principles adopted as a result “give legitimacy to NICE guidance” [36]
An *Ethics of Opportunity Costs (EOC)* is a substantive framework that further specifies AfR’s ‘relevance’ condition by stipulating that resources should be distributed with regard to allocative efficiency [40, 39]. Under EOC, technologies are judged primarily on their cost-effectiveness; that is, the amount of health they deliver per unit cost compared with available alternatives, measured by the so-called incremental cost-effectiveness ratio (ICER). An individual technology’s ICER is compared against NICE’s overall cost-effectiveness threshold: the point at which, theoretically, the health benefits displaced to fund a technology (the ‘opportunity cost’) exceed the health benefits that it can be expected to deliver. Maximising efficiency would therefore require the NHS to only adopt technologies whose ICERs fall below this threshold. However, under EOC wider equity concerns are also incorporated through the deliberations of NICE’s independent appraisal committees, which make allowances for other potentially relevant normative considerations. Relatively cost-ineffective technologies may thus be recommended if a committee judges these wider factors significant enough to justify the associated opportunity cost
NICE also refers in SVJ to Beauchamp and Childress’s ‘four principles’ of medical ethics (respect for autonomy, beneficence, non-maleficence and justice), stating that it “subscribes to [these] widely accepted moral principles” [36]. The AfR and EOC frameworks can be understood as comprising NICE’s specification of the fourth of these principles, justice, and therefore provide the practical basis for its implied claim that in acting in accordance with these frameworks it acts in a way that can be accepted as legitimate and fair

This approach was, until recently, publicly articulated in a 2008 document entitled ‘Social value judgements: Principles for the development of NICE guidance’ (hereafter ‘SVJ’) [36]. Since 2008, however, NICE’s methods have evolved significantly, raising questions about the extent to which they continue to align with the approach articulated in SVJ. A previous study by this author that empirically examined normative changes to NICE’s approach highlighted two key findings [2]. First, it showed that while NICE’s independent appraisal committees continue to reach their recommendations through a deliberative process, the substantive basis of these decisions has become more formalised over time, with NICE providing its committees with increasingly specific advice on how to respond to normative concerns. In particular, the study highlighted the emergence of several decision-rules “which limit committees’ potential to exercise judgement” in response to ethically challenging cases, while facilitating the recommendation of relatively cost-ineffective technologies in order to systematically prioritise the needs of particular groups (such as the terminally ill, or those suffering from rare conditions). Second, the study showed that NICE’s methods have promoted an increasingly generous view of what constitutes sufficient evidence to recommend a technology’s adoption, accelerating public access to new medicines but with reduced confidence about their likely impacts. The result, the study argued, is that NICE-recommended technologies are increasingly likely to displace more health than they deliver, undermining what NICE claims in SVJ is its foremost substantive goal: an efficient allocation of resources.

Since this study was initially published in 2019, SVJ has been succeeded by a new document: 'The principles that guide the development of NICE guidance and standards’ (hereafter ‘Principles’) [16, 17]. The primary aim of the current study is therefore to build on previous work by exploring how Principles updates NICE’s public articulation of its normative approach and brings it into alignment with current methods. In particular, given NICE’s reliance on the AfR framework—which conceives of publicity as a necessary condition of procedural fairness and legitimacy (Table 1)—this study examines whether Principles offers a transparent account of NICE’s approach and whether NICE therefore fulfils this aspect of its own specification of a fair decision-maker. It is also hoped that the study will provide useful context for future work concerned with the normative basis of NICE decision-making, which is currently undergoing further revision as the result of a major review of NICE’s approach and which continues to be a source of significant academic, public and political interest [18].^1^

## Methods

The study used documentary analysis to compare Principles with SVJ, and with NICE’s current methods as established by the 2019 study [2]. It consisted of three separate analyses.

First, a quantitative content analysis was performed across four texts: (1) the final Principles document, published in January 2020 [16]; (2) the draft Principles document, issued for consultation in November 2019 [19]; (3) the first edition of SVJ, published in 2005 [35], and (4) the second edition of SVJ, published in 2008 [36]. This analysis was designed to explore how Principles differs from SVJ in content, with the draft Principles document included to provide further insight to NICE’s evolving conceptualisation of its approach. In particular, this analysis was designed to test the hypothesis that Principles presents a more procedurally focused articulation of NICE’s approach, potentially undermining transparency about the actual substantive basis for NICE’s recommendations [12]. The content analysis was conducted according to the technique set out by White and Marsh [14]. Text was initially coded as either procedural, substantive or contextual, with eighteen further deductively-derived sub-codes used to provide a more granular understanding of the content included within each of these main categories. (Appendix 1). Simple descriptive statistics were employed to identify and present key findings. Coding was conducted by a single researcher; however, intracoder reliability was checked by re-coding two of the four documents several weeks after initial coding. The agreement rate was 94%, which was deemed acceptable for the purpose of the analysis.

Second, a comparative structural analysis was conducted across the same documents, with a focus on how different types of content are dispersed across two hierarchal levels: headline principles (indicated by bold type, large font, numbering and other signs of emphasis) and supporting text.

Finally, a targeted qualitative content analysis was undertaken to establish the extent to which Principles acknowledges and provides justification for the specific methodological changes highlighted by the 2019 study. This entailed collating relevant material from Principles and comparing it with data extracted as part of previous work. (A list of the documents included in this prior analysis is provided as Appendix 2.)

## Results

The analysis reveals significant overlap in the content of SVJ and Principles. Indeed, analysis indicates that despite the observed changes to NICE’s methods since 2008 [2], Principles derives much of its material directly from SVJ and contains relatively little new information to acknowledge or explain this updated approach. Existing content has, however, been substantially restructured. The analysis also confirms that Principles is considerably more procedurally focused than its predecessor and contains relatively little information on either the current substantive basis for NICE’s recommendations or the overall normative scheme on which NICE’s value judgements are based.

### Quantitative Content Analysis

**Fig. 1:**
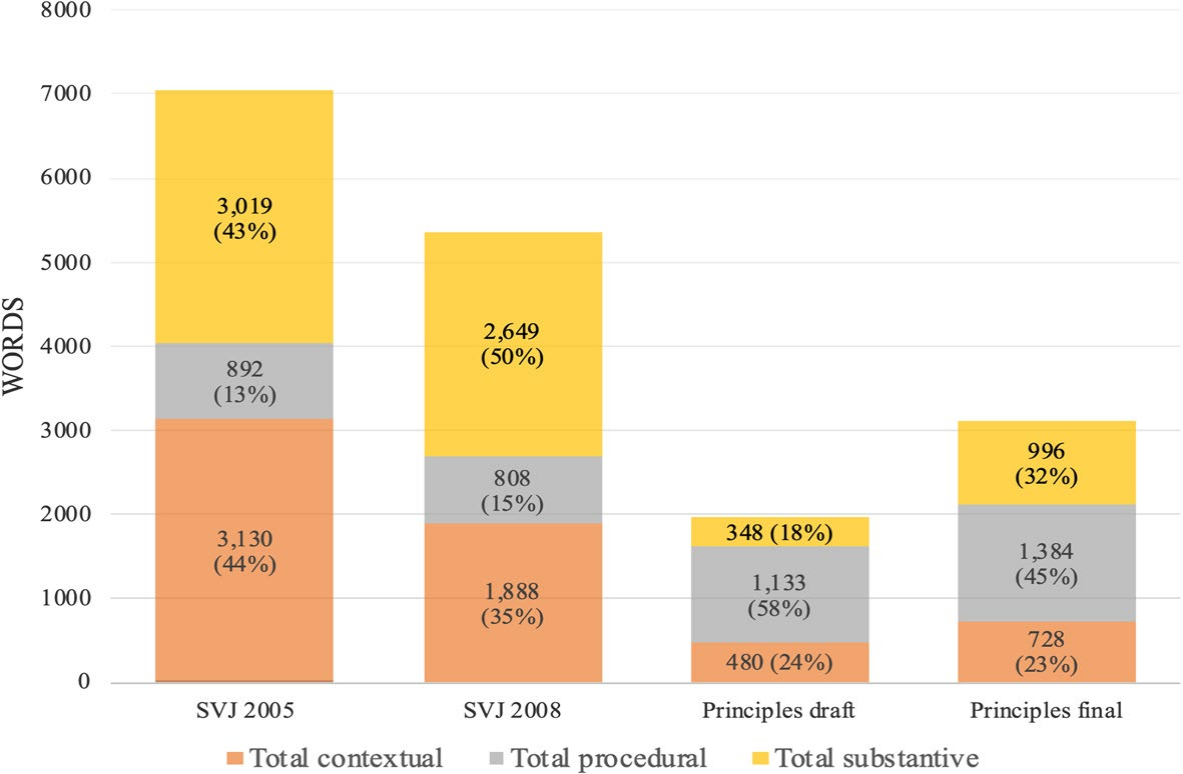
Overview of quantitative content analysis

One very obvious difference between SVJ and Principles is their relative length, which decreased from 7041 words in the first edition of SVJ to 3,108 words in the final version of Principles. (The draft version of Principles is particularly short, at only 1961 words) (Fig. 1). This absolute reduction in length is not, however, reflected equally across all categories of content, with Principles containing significantly more procedural content than its predecessor in both relative and absolute terms: while the two editions of SVJ contain 13% and 15% procedural content respectively, this figure increases to 45% in the final version of Principles (and to 58% in the draft version). Conversely, the proportion of substantive content decreases from 43% and 50% in the two editions of SVJ, to 32% in the final version of Principles (18% in the draft), while contextual content is similarly decreased from 44% and 35% in SVJ, to 23% in the final version of Principles (24% in the draft). Overall then, while procedural content comprises a relatively small part of SVJ, it is the dominant form of content in both the draft and final version of Principles.

**Fig. 2:**
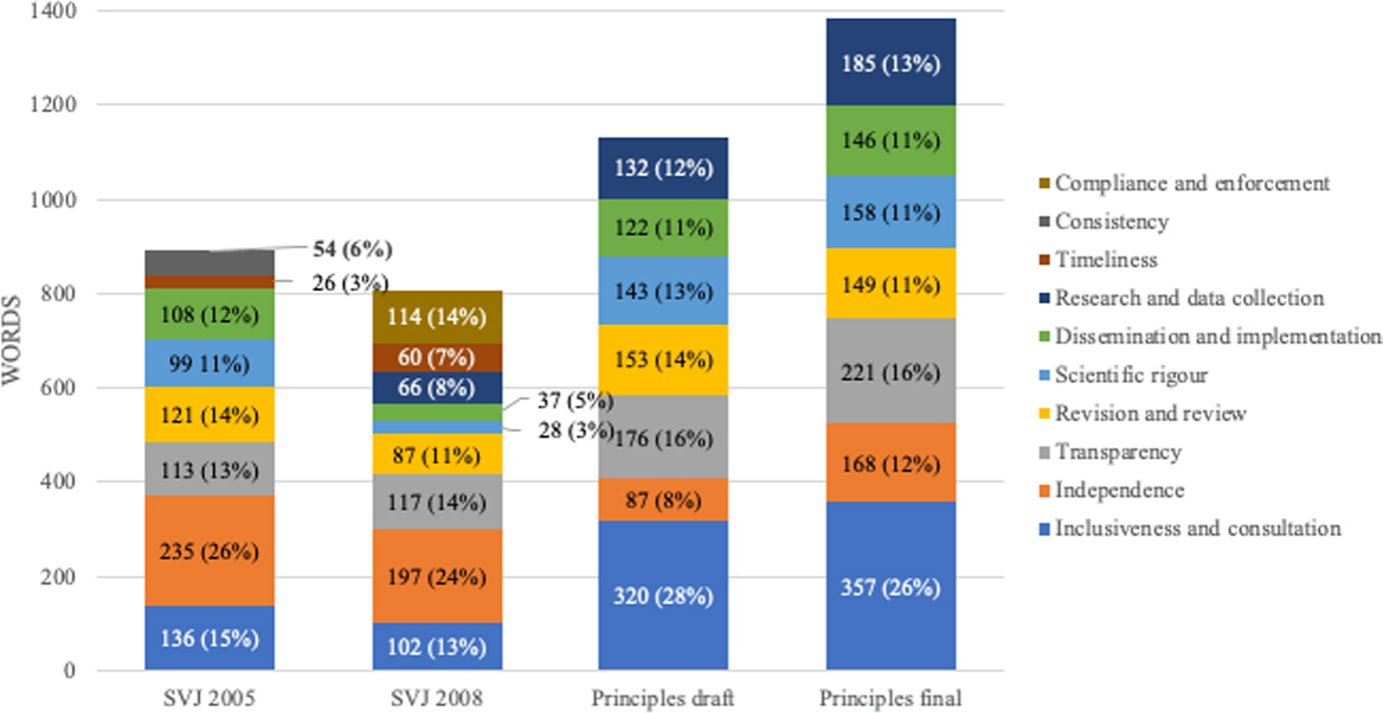
Breakdown of procedural content

Notable shifts are also evident within each of these main categories. Perhaps unsurprisingly given the absolute increase in procedural content, most procedural sub-categories are presented at greater length in Principles than in SVJ. However, in relative terms, there is a clear shift away from content relating to the independence of NICE’s processes and towards content relating to inclusiveness and consultation (Fig. 2). Transparency remains a consistent theme in both sets of documents, but consideration of scientific rigour, revision and review of guidance, research and data collection, and the dissemination and implementation of NICE’s advice is relatively increased in Principles compared with SVJ. Conversely, content relating to the timeliness of NICE’s advice, the need for consistency in NICE’s processes and methods, and procedures for ensuring compliance and enforcement of the principles contained within SVJ are absent from both versions of Principles. Despite the absolute increase in procedural content, Principles introduces no new procedural sub-categories and each of the procedural features presented in Principles is also described to some extent in SVJ.

**Fig. 3:**
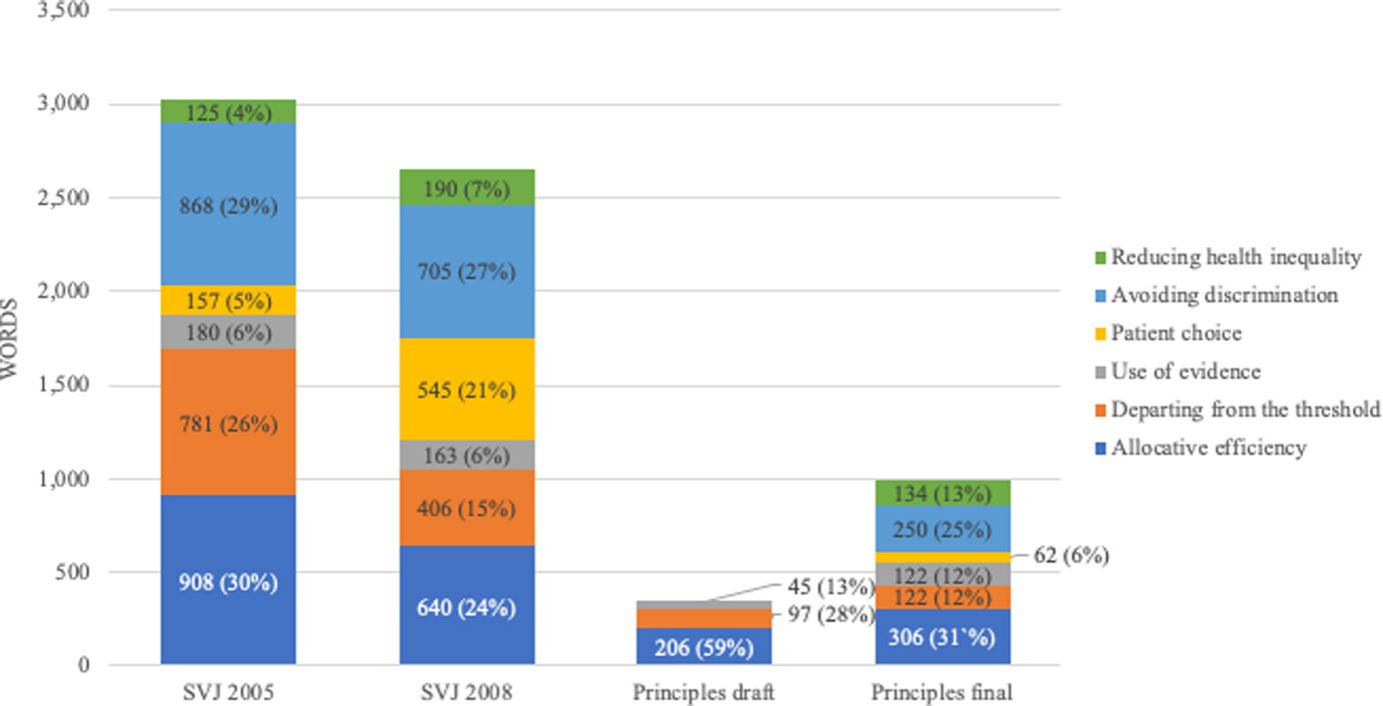
Breakdown of substantive content

For substantive content, the distribution across different sub-categories has remained relatively consistent, with matters relating to allocative efficiency (that is, cost-effectiveness) and the avoidance of unfair discrimination dominating (Fig. 3). However, given the absolute reduction in substantive content, Principles addresses these issues very briefly, with the final version devoting only 306 words to the concept of allocative efficiency, despite this traditionally being NICE’s main substantive goal (Table 1). (The equivalent figure in the draft version is 206 words). Other substantive considerations such as patient choice, the desire to reduce health inequality, the use of evidence, and the circumstances in which the usual cost-effectiveness threshold might be exceeded (‘modifiers’) are all covered in less than 150 words in the final version of Principles, with most of these topics unmentioned in the draft.

**Fig. 4:**
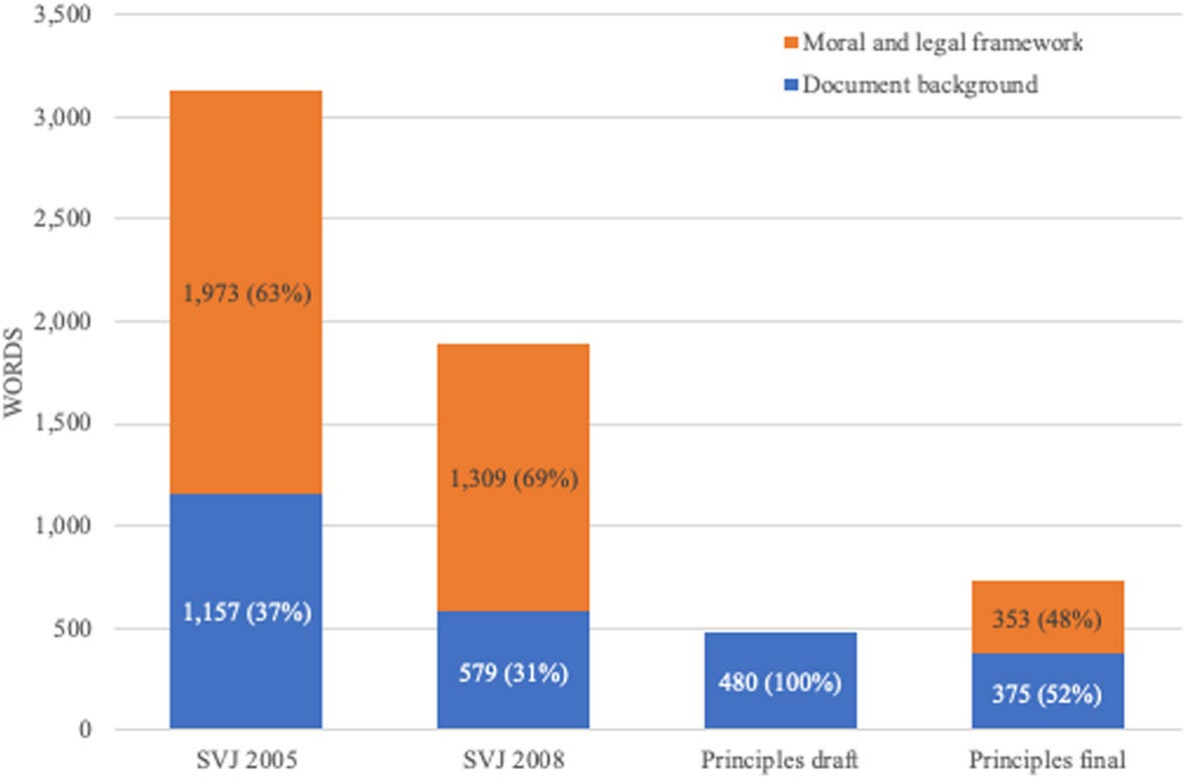
Breakdown of contextual content

The nature of the contextual content provided alongside the substantive and procedural aspects of NICE’s approach has also undergone notable change (Fig. 4). In both editions of SVJ, general background information—about NICE’s role, the document’s aims and so on—makes up around a third of this content, with the remainder consisting of information about NICE’s overall normative scheme (as outlined in Table 1). In the draft version of Principles, this normative material is completely absent, with the small amount of contextual content consisting entirely of general background information. In the final version, reference to three “moral principles” has been added, increasing normative content (Table 2). However, the document does not offer any information about how these principles have been derived or how they relate to the document’s other content. There is also ambiguity about the continued status of the normative scheme set out in SVJ. While the final version of Principles makes clear that it is intended to “replace” SVJ, it also states that “the original social value judgements document remains relevant to [NICE’s] work”, implying that Principles rests on similar normative foundations. However, unlike SVJ, Principles does not make any explicit reference to these foundations, leaving their formal status as an aspect of NICE’s approach unclear.^2^

**Table 2:** NICE’s current moral principles

In setting out the moral foundations for NICE’s approach, Principles [16] states the following:
NICE’s advisory committees use their own discretion when developing guidance and standards. But their decisions are guided by the principles in this document, which are based in part on the following moral principles:
People have the right to make informed choices about the care they receive. But not everyone has the ability to make their own choices, and not everything people might want will necessarily be available
Every intervention has the potential to cause harm and may not always benefit everyone. So it is important [to] consider the balance of benefits and harms when deciding whether an intervention is appropriate
Resources need to be allocated appropriately and fairly. They must provide the best outcomes for the finite resources available while balancing the needs of the overall population and of specific groups

### Structural Analysis

**Fig. 5:**
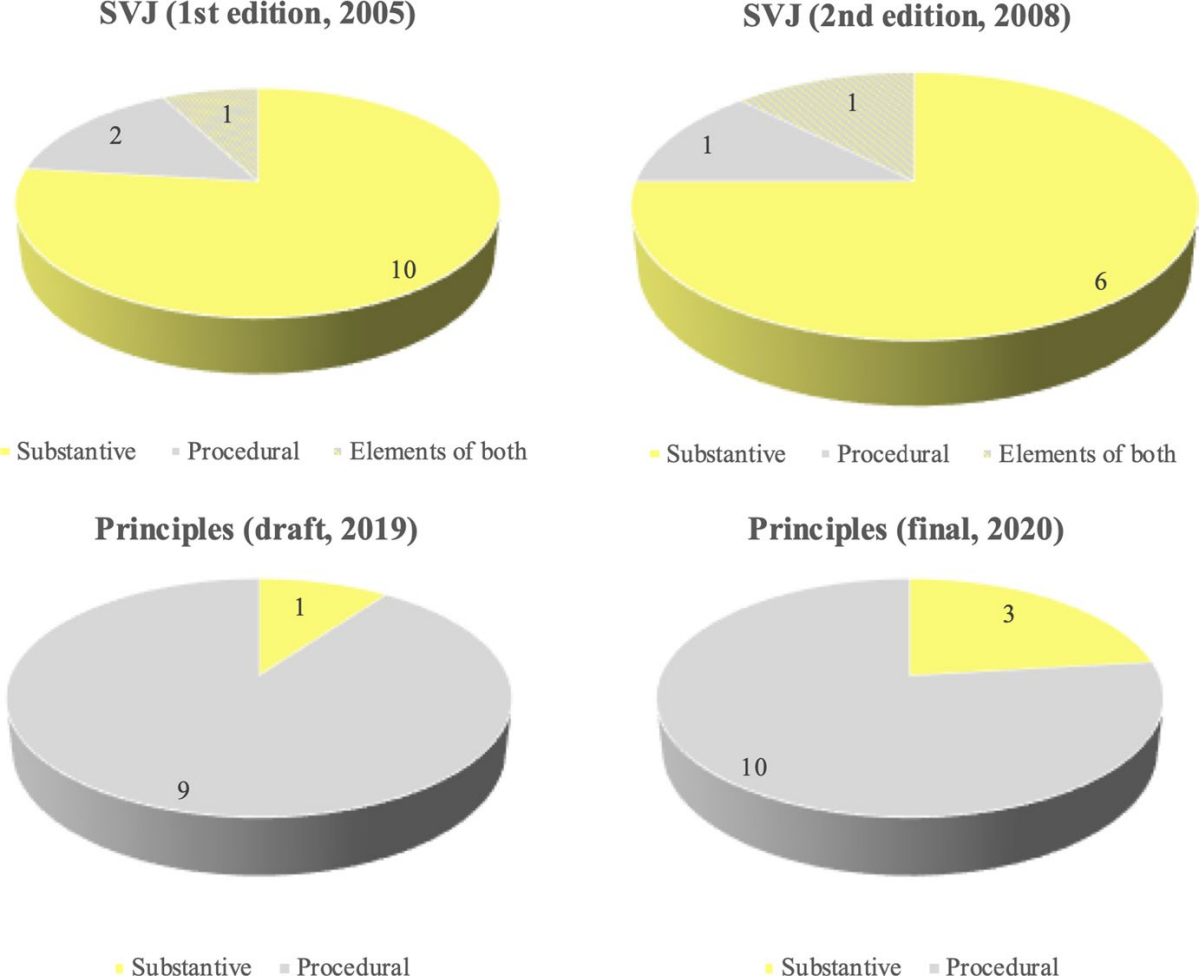
Headline principles by document, substantive versus procedural

The shift in focus towards procedural content is also evident from the structural analysis. In both editions of SVJ, most headline principles relate to substantive aspects of NICE’s approach, with procedural aspects set out as part of the preamble to these key principles. In the new document, however, several procedural features are ‘upgraded’ to standalone headline principles, while certain substantive principles are ‘downgraded’ to supporting text (Fig. 5 and Appendix 3).

For example, while the second edition of SVJ states as headline substantive principles both that NICE’s recommendations “must take into account the relative costs and benefits of interventions (their ‘cost-effectiveness’)” (Principle 2) and that they should incorporate consideration of “other factors” (Principle 3), the new document downgrades the latter to supporting text, effectively presenting it as subordinate to the principle of cost-effectiveness rather than as a distinct commitment carrying parity of esteem. Conversely, procedural commitments to transparency (Principle 2), independence (Principle 3), the dissemination and implementation of guidance (Principle 12) and the procedures through which guidance is reviewed and updated (Principle 13) are all based on content ‘upgraded’ from supporting text in SVJ. In addition, two further substantive principles derived from SVJ have been reframed in Principles in procedural terms. That is, SVJ’s Principle 1—which states that committees “should not recommend” an intervention if there is insufficient evidence on which to make a clear decision—has become a commitment to “use evidence that is relevant, reliable and robust”: a procedural assurance about how evidence will be employed rather than a substantive pledge not to base recommendations on weak evidence. Similarly, SVJ’s Principle 7—which states that “NICE can recommend that use of an intervention is restricted to a particular group of people” in certain circumstances—is replaced by a promise to “consider whether it is appropriate” to vary recommendations by group, committing NICE to following a particular procedure but not to any specific substantive outcome.

Thus, while the quantitative content analysis demonstrates a shift from substantive content in SVJ to procedural content in Principles, the structural analysis shows that this is mirrored by a shift from primarily substantive headline commitments in SVJ, to primarily procedural headline commitments in Principles.

### Qualitative Content Analysis

#### Decision-Rules

The 2019 study related how NICE’s deliberative approach to decision-making has, over time, been tempered by its increasing adoption of decision-rules that “seek to define normatively relevant considerations and guide committees’ response to them” [2] (Table 3.) A further aim of this study was therefore to establish the extent to which Principles acknowledges and provides justification for the value judgements embedded in these decision-rules.

**Table 3:** Current NICE decision-rules as identified by Charlton, 2019

The previous study identified four decision-rules that are used under NICE’s current methods to define normatively relevant considerations and guide appraisal committees’ response to them [2]:
The use of a *specified cost-effectiveness threshold range* of £20,000-£30,000 per quality-adjusted life-year (QALY), to indicate whether a technology should generally be considered to offer acceptable value-for-money
The so-called *‘end-of-life’ (EOL) criteria*, which in effect increase this threshold to £50,000/QALY for technologies offering an extension to life of at least three months, in patients with a life expectancy of less than 24 months
The use of a *£100,000/QALY threshold for drugs assessed via the highly specialised technologies (HST) programme*, further enhanced to up to £300,000/QALY where the magnitude of benefit is particularly large, and
The use of a *lower discount rate* (1.5% vs 3.5%) for technologies that offer large health gains over a long time period, effectively leading to a more generous cost-effectiveness estimate

Three of the four identified decision-rules were established after the publication of the second edition of SVJ in 2008; they are therefore not referred to in either version of this document. However, these rules also go largely unmentioned in Principles, despite pre-dating its publication. (Table 4). In the draft version of Principles, brief reference is made to the so-called end-of-life (EOL) criteria, which prioritise health benefits accruing to terminally ill patients according to certain conditions.^3^ However, in the final version, this reference has been removed. A similarly brief allusion in the draft version to the highly specialised technologies (HST) programme—and its recommendation of technologies with ICERs “above our normally acceptable range”- is retained in the final version, but with a change in wording that substitutes explicit acknowledgement of the programme’s higher threshold with a more ambiguous indication that “a *different* threshold is applied” [emphasis added] for these technologies. None of these references acknowledge the magnitude of uplift to the cost-effectiveness threshold for EOL and highly specialised technologies to around £50,000 per Quality-Adjusted Life-Year (QALY) and £100,000–£300,000/QALY respectively [20, 21] or, in the case of HSTs, to the use of a QALY weighting formula to prioritise technologies based on their potential magnitude of health benefit. Neither document makes any reference to NICE’s acceptance of a lower discount rate for technologies that offer large health gains over a long time-period: a decision-rule that effectively prioritises the needs of young people suffering from very severe, life-limiting conditions [22, 37, 38].^4^

**Table 4:** Presentation of decision-rules in SVJ and Principles

	SVJ, 2005	SVJ, 2008	Draft Principles, 2019	Final Principles, 2020
Cost-effectiveness threshold (established c.2004)	Principle 5: NICE guidance should explain, explicitly, reasons for recommending – as cost effective – those interventions with an incremental cost-effectiveness ratio in excess of £20,000 to £30,000 per QALY	Principle 4: NICE should explain its reasons when it decides that an intervention with an ICER below £20,000 per QALY gained is not cost effective; and when an intervention with an ICER of more than £20,000 to £30,000 per QALY gained is cost effective	No direct reference	Interventions with an ICER of less than £20,000 per QALY gained are generally considered to be cost effective. Our methods manuals explain when it might be acceptable to recommend an intervention with a higher cost-effectiveness estimate
EOL criteria (established 2009)	Rule not yet introduced		For treatments that extend life for people at the end of life, and for highly specialised technologies where specific criteria are satisfied, we may recommend an intervention with a cost-effectiveness estimate above our normally acceptable range	No direct reference
QALY weighting for HSTs (established 2017)	Rule not yet introduced			A different threshold is applied for interventions that meet the criteria to be assessed as a ‘highly specialised technology’
Lower discount rate in special circumstances (established 2011)	Rule not yet introduced		No direct reference	No direct reference

Principles is also more ambiguous than its predecessor about the cost-effectiveness threshold itself. Although SVJ does not explicitly state that NICE applies a threshold in its approach to decision-making, it acknowledges that “in general, interventions with an ICER of less than £20,000 per QALY gained are considered to be cost effective” and that “as the ICER of an intervention increases in the £20,000–£30,000 range, an advisory body’s judgement about its acceptability” should make reference to reasons aside from cost-effectiveness. In contrast, the draft version of Principles makes no reference to this £20,000–£30,000 threshold range, stating only that “the way that we assess value for money is set out in detail in our method manuals”. During consultation, this ambiguity about the threshold was interpreted by some as “a deliberate decision” to give NICE “greater scope for departing from [it]” [23]. In response, NICE assured consultees that “an existing principle has been expanded to explain that NICE’s core method for assessing value for money is cost-effectiveness analysis and that our standard threshold is £20,000–£30,000” [23]. However, the final document in fact refers only to the lower bound, stating that “interventions with an ICER of less than £20,000 per QALY gained are generally considered to be cost effective” and that “our methods manuals explain when it might be acceptable to recommend an intervention with a higher cost-effectiveness estimate”. Principles, like SVJ, also does not offer any clear empirical or normative rationale for the £20,000–£30,000/QALY range, stating only that it “takes into account the ‘opportunity cost’ of recommending one intervention instead of another”. It does not offer any specific reasons for NICE’s willingness to accept greater opportunity cost in certain circumstances.

Thus of the four decision-rules considered, only two are acknowledged in Principles: NICE’s use of a cost-effectiveness threshold and its adoption of a different threshold for HSTs. In both cases, important substantive elements of the decision-rule are omitted and, in the case of HSTs, Principles offers no reason for NICE’s prioritisation of these technologies. Indeed, Principles continues to state as its main substantive commitment that NICE’s recommendations will be based on “an assessment of population benefits and value for money”. As the selected examples detailed in Table 5 illustrate, the reality of NICE decision-making is often much more nuanced and can involve balancing a much wider range of value judgements than this commitment implies.

**Table 5:** Selected examples of recent NICE recommendations

Pertuzumab with trastuzumab and docetaxel for treating HER2-positive breast cancer
Pertuzumab (Perjeta) is a monoclonal antibody administered by intravenous infusion that targets human epidermal growth factor receptor 2 (HER2). It is indicated in combination with trastuzumab (Herceptin) and docetaxel for the treatment of patients with HER2 positive breast cancer. Prior to NICE’s recommendation of pertuzumab in 2018, the treatment had been available to NHS patients for several years via the Cancer Drugs Fund. This contributed to an evidence base which, by 2018, the NICE appraisal committee considered to “clearly demonstrate[s] that the addition of pertuzumab to trastuzumab leads to a substantial improvement in progression-free and overall survival, which is unprecedented in the treatment of advanced breast cancer” [31]. As such, the committee stated that it “considered the appraisal of pertuzumab to be a special case”, explaining that there was a need to be mindful of “removing funding for an effective treatment which has become, in the minds of patients and clinicians, standard of care for treating metastatic breast cancer” [31]. The committee therefore chose to exercise “flexibility” in its application of the end-of-life (EOL) criteria, concluding that “it was fair and reasonable to accept that pertuzumab fulfilled the criteria for special consideration,” even though life expectancy for this patient group exceeded 24 months [31]. In addition, the committee appears to have been willing to recommend pertuzumab at an ICER beyond the £50,000/QALY threshold usually applied under the EOL criteria, stating that “in the context of the exceptional circumstance this case presents, it would be reasonable not to be asked to have to apply the maximum weight to the QALYs gained by pertuzumab” [31]. The actual ICER used in decision-making was classified as commercial in confidence and was not made public. However, the committee acknowledged that “even if the company’s base-case ICER was accepted as plausible, there was a 0% probability that pertuzumab plus trastuzumab and docetaxel could be considered cost effective at a maximum acceptable ICER of £30,000 per QALY gained” [31]
Naltrexone–bupropion for managing overweight and obesity
Naltrexone–bupropion (Mysimba) is a prolonged-release weight-loss tablet indicated for obese and overweight adults. It was evaluated and rejected through NICE’s core technology appraisal programme in 2017 [32]. Although the drug’s estimated ICER of £23,750/QALY was within the range generally considered to be an acceptable use of NHS resources, the committee highlighted that “the patient population is potentially very large and treatment is long-term”, leading to a potentially “high impact on NHS resources”. It therefore concluded that it “needed to be certain that naltrexone–bupropion will provide value to the NHS” [32]. As such, although the committee recognized that “new pharmacological treatment options to manage overweight and obesity are needed” and that naltrexone–bupropion appeared to be somewhat effective in reducing weight, it concluded that it was “unable to recommend naltrexone–bupropion as a cost-effective treatment for use in the NHS” because of “considerable uncertainty about the true ICER” [32]
Cerliponase alfa for treating neuronal ceroid lipofuscinosis type 2 (Batten disease)
In November 2019, NICE’s highly specialised technologies (HST) committee recommended the enzyme replacement therapy cerliponase alfa (Brineura) for the treatment of neuronal ceroid lipofuscinosis type 2 (CLN2), an inherited condition that leads to rapid physical and mental decline in young children and typically death by early adolescence. In reaching this decision, the committee “considered that CLN2 is a rare, devastating condition, with a debilitating and life-limiting effect on children […] and that it has a substantial emotional and financial impact on their families” [33]. It further recognized that “there was an unmet need for an effective treatment” and that “cerliponase alfa is innovative and represents an important development in treating the condition” [33]. The committee acknowledged that the treatment’s long-term effects are “associated with substantial uncertainty” and that “the company's original assumptions around disease stabilisation, mortality and starting distribution were unrealistic” [33]. Nevertheless, taking into account its preferred assumptions, the committee agreed that cerliponase alfa met the criteria for a QALY weight of 3.0, uplifting the cost-effectiveness threshold to £300,000/QALY, the maximum acceptable level under NICE’s current methods. The committee concluded that “while highly uncertain, it was plausible” that the ICER of cerliponase alfa was “within the range normally considered an effective use of NHS resources for highly specialised technologies” (that is, £100,000–£300,000/QALY) [33]. The actual estimated ICER was judged to be commercially sensitive and was therefore not made public

The above examples and the extracts presented have been purposively selected to illustrate the nuanced nature of NICE decision-making and the wide range of value judgements that appraisal committees invoke in reaching their decisions. It is not intended to and does not offer a representative picture of NICE decision-making as a whole

#### NICE’s Approach to Evidence-Based Decision-Making

A further finding of the 2019 study was NICE’s increasingly generous view of what constitutes sufficient evidence to recommend a technology’s adoption, accelerating access to new medicines but with reduced confidence about their likely impacts [2]. The final aim of this study was therefore to explore the extent to which Principles acknowledges and explains this evolution in NICE’s approach to evidence-based decision-making.

Historically, NICE has expressed a clear preference for data derived from the highest levels of the so-called ‘hierarchy of evidence’, with other data primarily used to “supplement” the results of randomised controlled trials (RCTs) [24]. This insistence on robust evidence is strongly reflected in the second edition of SVJ, which states as Principle 1 that “committees should not recommend an intervention if there is no evidence, or not enough evidence, on which to make a clear decision”. Today, although NICE retains its preference for RCTs, its methods indicate an increased willingness to recommend technologies based on other study types, despite these being associated with greater uncertainty [2]. Accordingly, Principles states that while NICE continues to “recognise the value of traditional ‘hierarchies of evidence’”, it takes “a comprehensive approach to assessing the best evidence that is available”. Nevertheless, the document repeats verbatim SVJ’s claim that technologies will not be recommended “if there is no evidence, or not enough evidence, on which to make a clear decision” and states as a headline principle that NICE will base its decisions on evidence that is “relevant, reliable and robust”, strongly implying that demanding evidential standards continue to be applied across NICE’s activities.

NICE’s methods, however, stipulate several circumstances in which this is not the case. In considering technologies indicated for rare diseases, for example, appraisal committees are advised that “the evidence base will necessarily be weaker” than normal and that this should be taken into account [22]. Similarly, NICE’s methods formally place lower evidential requirements on cancer drugs, both through the EOL criteria and NICE’s operation of the cancer drugs fund (CDF).^5^ In fulfilling the criterion that EOL technologies should offer at least a three-month extension to life, NICE requires only that a technology offers “the prospect of” delivering the required benefit, estimates of which can be either shown directly or “reasonably inferred” from surrogate endpoints such as progression-free survival [20]. Moreover, in cases in which “the uncertainty […] is too great to recommend the drug for routine use” even under these more generous standards, a cancer drug must only “display plausible potential” for satisfying NICE’s usual cost-effectiveness criteria to gain conditional approval via the CDF [20]. This effectively reverses the burden of proof in these cases by requiring appraisal committees to demonstrate that a technology is *not* cost-effective in order to reject it [2].

Although CDF recommendations typically carry requirements for further data collection and Principles acknowledges the use of such arrangements to “resolve uncertainties in the evidence”, it is not explicit about NICE’s willingness to recommend technologies in the absence of clear evidence. Nor is it open about the different evidential standards applied to different technology types (Table 5), or its practical or normative reasons for adopting these. Thus, the straightforward commitment made in Principles to base recommendations on evidence that is “relevant, reliable and robust” does not fully reflect the increasingly nuanced and flexible approach to evidence that NICE now adopts in practice.

## Discussion

NICE’s approach to priority-setting has evolved significantly since 2008, with its methods incorporating an increasingly complex set of substantive value judgements that together imply greater willingness to recommend technologies that likely displace more health than they deliver [2]. Given these changes, and given NICE’s commitment to transparency, it might have been anticipated that Principles would differ markedly from its predecessor. In particular, given NICE’s historical focus on allocative efficiency as its primary distributive concern, Principles might have been expected to either highlight and justify NICE’s adoption of formal rules and norms that are in tension with this, or to set out how and why allocative efficiency is no longer its main substantive objective.

This study demonstrates that Principles does not fulfil this expectation. Despite the evolution of NICE’s methods, Principles derives much of its headline content directly from SVJ, incorporating little new information to acknowledge or explain its updated approach and omitting much of the foundational material that previously tethered NICE’s principles to an underlying normative framework. Principles also provides a procedurally-focused account of NICE’s approach at a time when its methods are becoming increasingly reliant on formal substantive rules and ‘modifiers’ that cannot be justified in purely procedural terms [2]. Thus, while Principles tells NICE’s stakeholders much about how the organisation goes about the process of decision-making, it tells them little about the substantive grounds on which its decisions are now based.

Setting aside the question of whether these grounds are ethically sound, it will be argued here that NICE’s failure to fully acknowledge and explain them in Principles undermines the fairness and legitimacy of its decision-making. This argument will proceed as follows. First, it will be shown that a commitment to transparency remains central to NICE’s understanding of procedural justice and its specification of a fair decision-maker. Second, it will be argued that any reasonable conception of transparency requires that the account offered be sufficiently complete for stakeholders to understand and engage with the main grounds for decision-making. Third, it will be shown that Principles, as a standalone document, fails to meet this requirement because it does not acknowledge the main grounds for decision-making and does not explain its reasons for adopting those grounds that it does acknowledge. Fourth, it will be shown that while other documents supplement the information provided by Principles, the account provided by this body of documentation remains incomplete and is also inaccessible in practical terms. Thus, it will be argued that NICE does not currently provide a transparent account of its approach and, given its continued reliance on transparency as a requirement of procedural justice, that it therefore does not currently fulfil this aspect of its own specification of a just decision-maker. Finally, brief consideration will be given to why NICE might currently find it difficult or impossible to meet the demands of transparency in this domain.

### Transparency as a Condition of Fairness

NICE’s approach to justice has historically relied on compliance with the requirements of Daniels and Sabin’s AfR framework. One such requirement is publicity: the need for both priority-setting decisions and the grounds for reaching them to be “publicly accessible” [7, 35, 36]. The need for publicity derives from the lack of societal consensus about how to allocate scarce resources and the legitimacy problem that arises when such decisions are, by necessity, made on society’s behalf. According to AfR, this is best addressed by being open about “the facts, reasons and principles that are relevant to the dispute”, thereby facilitating “public deliberation and democratic oversight” [8]. As Daniels puts it: “There must be no secrets where justice is involved, for people should not be expected to accept decisions that affect their wellbeing unless they are aware of the grounds for those decisions” [6].

Unlike SVJ, Principles does not explicitly refer to AfR or formally subscribe to the publicity requirement. However, it restates NICE’s commitment to transparency in similar terms, noting the “significant impact” that NICE’s recommendations can have on people’s lives and the need for them to be based on “a process that is transparent and contestable” in order to maintain “credibility” [16]. Thus, although the current status of AfR as a formal component of NICE’s approach is ambiguous, transparency remains central to its conception of procedural justice and Principles, like SVJ, commits NICE to being open about the reasons for its recommendations.

### The Demands of Transparency in Practice

Accepting that NICE’s conception of procedural justice requires that the grounds for decision-making be made public, the question turns to what this demands in practice. According to AfR, publicity does not imply that “all criteria for decision-making be set in advance or explicitly agreed upon ahead of time” [8]; it could therefore be argued that as long as a priority-setter is transparent about its reasons for reaching individual recommendations, there is no need for it to standardise these and describe them within a general account of its approach. However, given that NICE *has* chosen to standardise its reasons (as indicated by its use of increasingly detailed process and methods guides) and given that Principles claims to offer an account of the “morals, ethics and values” underpinning NICE’s approach [16], it is reasonable to expect that this account will be sufficiently detailed to cover the main substantive grounds for decision-making.

One scenario in which a full description of these grounds might not be in the interests of transparency is if such an account would be inherently inaccessible, for example due to its length or complexity. However, this scenario seems unlikely. Even very complex concepts can generally be made accessible if skilfully communicated, and there is no reason why an ethically coherent approach to priority-setting would be so complicated as to preclude comprehension by someone invested in understanding it. Indeed, it could be argued that such an approach would be fundamentally incompatible with AfR, because it makes open deliberation about the grounds for decision-making impossible. However, even if one accepts that a balance must be found between completeness and accessibility, then any partial account of the grounds for priority-setting must still be capable of fulfilling the basic requirement of transparency; that is, it must provide enough information for stakeholders to engage with the key “facts, reasons and principles” that underpin decision-making [8].

### Transparency and the Principles Document

NICE implies that Principles fulfils this requirement, describing it as a resource intended to “help anyone interested in NICE better understand what we take into account when developing our guidance” [17]. However, it does not claim that Principles is a complete account of NICE’s normative approach; rather, it acknowledges that its scope is limited to “the key principles that apply across all our guidance and standards” [17]. In limiting its scope in this way, however, Principles omits several important grounds for NICE’s recommendations. The EOL criteria are not applied to every technology that NICE appraises, nor are they relevant to every programme; nevertheless, they are a general feature of NICE’s approach and should therefore, according to the publicity condition, be open to “public deliberation and democratic oversight” [8]. Deliberate exclusion of such grounds on the basis that they are not ‘key’ or universal undermines transparency. Such exclusions also prevent stakeholders from scrutinising and challenging potential inconsistencies in NICE’s normative approach. For example, NICE has historically taken the position that drugs to treat rare conditions do not generally warrant prioritisation and should be evaluated “in the same way as any other treatment” [36]. However, the HST programme is specifically designed to recognise and respond to “the vulnerability of very small patient groups”, granting significant priority to drugs for very rare diseases [35] (Table 5). Because neither of these value judgements is universal, Principles by design excludes them, hindering public scrutiny of these seemingly inconsistent positions.

Even if Principles could restrict its scope to key universal principles without undermining transparency, there are indications that these criteria have not been consistently employed. The higher threshold applied to HSTs occurs in the context of a single appraisal programme, which to date has considered 15 technologies in eight years. In contrast, the EOL criteria are applied across multiple programmes and have been used dozens of times to justify recommendations. Yet Principles alludes to the former, while ignoring the latter. Similarly, the upper bound of the £20,000-£30,000/QALY threshold unacknowledged in Principles is fundamental to NICE’s determination of cost-effectiveness and has been shown in practice to more accurately predict the likelihood of recommendation than the (acknowledged) lower bound [5]. Principles (like SVJ) is also silent about another fundamental aspect of NICE’s approach: the perspective taken in assessing a technology’s impacts and NICE’s reasons for limiting its consideration to direct health effects and costs to the health system, while excluding wider societal benefits. Indeed, the draft Principles document is notable for its almost complete lack of substantive content, leading one group of consultees to argue that “the move away from substantive ethical values towards procedural principles enables the detail of [NICE’s] decision-making procedures and the content of recommendations to remain almost entirely unspecified” [12]. Though NICE sought to respond to such feedback by reinstating some material from SVJ post-consultation, the 996 words devoted to substantive content in the final version of Principles remains insufficient to describe the highly intricate grounds for decision-making established by NICE’s current methods.

Principles also does not fully articulate NICE’s reasons for adopting such grounds. Principles states that “a different threshold” is applied to HSTs, but does not explain why [16]. Nor does it explain why considerations about social stigma and lifestyle choices are generally excluded from decision-making, while consideration of “inequalities arising from socioeconomic factors and the circumstances of certain groups of people, such as looked-after children and people who are homeless” are included [16]. Principles highlights the importance that NICE places on “promoting innovation in the provision of health services” but does not give its reasons for differentially valuing health benefits arising from such innovation [3]. These omissions hinder public debate about NICE’s reasons for adopting such positions and, given Principles’ lack of normative contextual content, individual value judgements are left without justification, untethered to any specified moral principles or wider normative scheme.

Whether or not one agrees with these value judgements, they form crucial aspects of NICE’s approach and together comprise the grounds on which recommendations are generally based. As such, an account that fails to acknowledge or provide reasons for adopting such grounds cannot, in itself, be considered to meet the demands of transparency.

### Other Potential Sources of Transparency

An obvious rebuttal is that Principles is not intended to operate as a standalone document: NICE expressly states that it “should be read in conjunction with our methods and process guides and our charter” [23]. It could therefore be argued that although Principles does not *by itself* meet the demands of transparency, these demands are nevertheless met when considering the entirety of information made available by NICE.

There are four sources of information that might, alongside Principles, satisfy such demands. The first is the detailed documentation that accompanies each appraisal. This material unquestionably provides a rich source of information about the context in which individual recommendations are made and gives significant visibility over these decisions. (As, for example, in the cases described in Table 5). However, its accessibility is severely limited by its volume, often stretching to many hundreds of pages, and its technical complexity. Evidence also shows that recent changes to NICE’s methods have led to the grounds for decision-making becoming less evident in these documents, both because normative considerations are increasingly embedded in the assessment process and are therefore not a topic of open deliberation [2] and because the results of cost-effectiveness analysis (particularly for cancer drugs) now frequently go unreported due to confidential price discounts [44]. More fundamentally, this body of material is not a good source of information about NICE’s *general* approach. While a patient wanting to understand why a particular drug has been recommended or rejected will likely find this resource highly relevant and useful, someone with a broader interest in NICE’s approach would have to examine many appraisals to get a sense of the considerations that NICE *generally* takes into account. As such, this source does not fulfil the need for NICE to be open about the general basis for its recommendations.

A second option is NICE’s charter: “a statement of purpose that describes who we are, what we do and how we do it” [16]. This document offers only a brief overview of NICE’s approach, but nevertheless supplements the substantive information provided by Principles by acknowledging and specifying the various cost-effectiveness thresholds that NICE adopts [34]. However, like Principles, it says nothing about NICE’s reasons for adopting these different thresholds. The charter also contributes to a potentially misleading account of NICE’s approach by reproducing Principles’ 13 headline statements but without the context provided by the document as a whole. Thus, NICE’s complex relationship with evidence, for example, is reduced to the unqualified statement, “We use evidence that is relevant, reliable and robust” [34]. It is therefore not clear that the charter can be considered to enhance the transparency of NICE’s general approach.

A third source is the collection of programme-specific process and methods guides that, by definition, describe NICE’s formal approach. Principles repeatedly refers stakeholders to these documents to understand, for example, when different types of evidence might be deemed appropriate or when a technology costing more than £20,000/QALY might be recommended. However, this is a highly inaccessible source of information, consisting of multiple documents that together span many hundreds of pages of often extremely technical material.^6^ Although these documents are clearly written and well presented, normative content is not always clearly signalled and significant amounts of technical information must therefore be filtered and understood in order to identify the value judgements embedded within it.^7^ Thus, notwithstanding current efforts to consolidate these guides as part of NICE’s ongoing process and methods review, the volume and complexity of this material seems an unavoidable barrier to transparency. These documents also provide little or no information on NICE’s reasons for adopting certain value judgements. In the words of one group of consultees, therefore, “simply signposting interested parties to the technical and process manuals might be considered efficient, but it is analogous to inviting people to read the New Testament in order to identify key Christian values” [12].

Finally, there is SVJ itself. Principles states that “the original social value judgements document remains relevant to [NICE’s] work” and provides an online link to the second edition [16]. However, as previously discussed, SVJ’s formal status is ambiguous, making it unclear how the information that it presents should be interpreted and applied. More fundamentally, SVJ is a legacy document whose contents no longer reflect the approach established by NICE’s methods, hence (presumably) NICE’s decision to replace it.^8^

In conclusion, therefore, while these sources provide further detail about some of the substantive grounds for NICE decision-making, the account that they offer remains incomplete and is largely inaccessible in practical terms. As such, NICE cannot be considered to satisfy the demands of transparency and therefore does not currently fulfil this aspect of its own specification of a just decision-maker.

### Why does NICE not Provide a Transparent Account of its Normative Approach?

It is clear from NICE’s public statements that it is aware of its moral and social responsibilities as a priority-setter and recognises the importance of transparency as a mechanism for meeting these [11, 13, 15, 17, 35, 36]. As an organisation, it also has a reputation for acting with integrity in its efforts to improve the accessibility and inclusiveness of its decision-making and in its attempts to reflect societal views in its approach [9, 42]. Why, then, has it not succeeded in producing a transparent account of this approach in Principles?

The most generous interpretation is that NICE has acted in good faith in attempting to provide a transparent account, but that the complexity of its current normative approach is such that a complete account would be inherently inaccessible. The next best option is therefore to present an account that is accessible but incomplete. This interpretation is consistent with NICE’s claim that Principles offers a “simpler and more accessible” account than SVJ [23], but does not explain why important substantive details that could be communicated very simply, for example, the upper bound of the £20,000–£30,000/QALY threshold or the prioritisation of EOL treatments, have nevertheless been omitted. The implication that a complete account of the substantive principles embedded across NICE’s activities would be inherently inaccessible is also revealing because it implies the absence of a coherent underlying framework; if such a framework existed, then even a very wide range of value judgements could likely be placed within it and presented relatively simply. Conversely, transparently presenting NICE’s approach becomes extremely challenging if it has evolved in ways that undermine its overall coherence. A less generous interpretation is therefore that Principles intentionally focuses on those aspects of NICE’s approach that can be easily explained and justified within the general framework set out by SVJ, while overlooking elements that are in tension with this traditional approach. This interpretation appears to more adequately explain NICE’s emphasis on widely accepted procedural values, which continue to reflect the general requirements of AfR, and its apparent reluctance to provide a full account of the varied substantive criteria now embedded across its methods, several of which conflict with both NICE’s traditional concern with allocative efficiency and, potentially, with each other. Thus, according to this interpretation, while there may be sound organisational and political reasons for NICE’s design of the new Principles document, it is not and was never intended to be a transparent presentation of NICE’s current basis for decision-making.^9^

## Conclusion

The 2019 study argued that NICE’s methods have evolved in ways that reflect the Institute’s increased willingness to exact an opportunity cost on the NHS in order to prioritise the needs of particular patient groups [2]. Setting aside whether such changes are justified, it argued that this had created a disparity between NICE’s stated normative approach articulated, at the time, in SVJ and the approach established by its methods, undermining transparency and weakening the claim that NICE acts in a way that is procedurally just. If this argument is to be accepted, then the publication of Principles offered an opportunity for NICE to bring these two versions of its approach back into alignment and thereby enhance the fairness of its decision-making.

The results of this study, however, suggest that Principles was never intended to bridge this gap. Rather, in Principles, NICE appears to have intentionally produced a document that grounds its claims for fairness and legitimacy almost entirely on the Institute’s procedural strengths, eschewing acknowledgement of the more contentious substantive considerations that have recently become embedded within its methods. Its reasons for doing so are understandable: Principles likely represents NICE’s best effort at presenting a set of complex and potentially incompatible substantive judgements in a way that appears superficially coherent and is accessible to a wide audience. However, in attempting primarily to avoid rather than address underlying questions about these judgements, Principles undermines the transparency on which NICE’s notion of procedural justice relies.

The development of the Principles document represented an opportunity for NICE to either fully articulate its normative approach and thereby demonstrate its legitimacy to those whose lives NICE’s decisions effect, or to reconsider and reformulate aspects that might be deemed problematic were they made open to public scrutiny. A similar opportunity is provided by the ongoing process and methods review, which seems likely to mark a further important milestone in NICE’s evolution as a healthcare priority-setter. It might be hoped that the changes that result from this review will provide NICE with the confidence to articulate its approach more fully and transparently in the future and that Principles will be updated accordingly.

## Data Availability

The datasets generated during and/or analysed during the current study are available from the corresponding author on reasonable request.

## Appendix

### Appendix 1: Codes Applied in Content Analysis

- *Contextual*: Relating to general background information and material on the normative and legal frameworks that provide the basis for NICE’s approach
  - Document background
  - Moral and legal framework
- *Procedural*: Content relating to procedural features of NICE’s approach; that is, features that ought to be present in the process of priority-setting.
  - Inclusiveness and consultation
  - Independence
  - Transparency
  - Challenge, revision and review
  - Scientific rigour
  - Dissemination and implementation of guidance
  - Research and data collection
  - Consistency of process and methods
  - Timeliness of advice
  - Compliance and enforcement of process and methods
- *Substantive*: Content relating to substantive features of NICE’s approach; that is, normative criteria or reasons that ought to inform priority-setting decisions and recommendations.
  - Allocative efficiency (i.e. cost-effectiveness as a basis for decision-making)
  - Departing from the threshold/’modifiers’
  - Evidence as a basis for decision-making
  - Patient choice and autonomy
  - Discrimination
  - Health inequality

### Appendix 2: Documents Included in Systematic Review of Policy, Reported in Charlton 2020 [2]

**Table:**

Year	Key policy documents*	Supporting documents
1999	Appraisal of new and existing technologies: Interim guidance for manufacturers and sponsors	
2001	Guide to the technology appraisal process (1st ed.) Guidance for manufacturers and sponsors/Guide to the methods of technology appraisal (1st ed.) Guidance for appellants	Guidance for healthcare professional groups Guidance for patient/carer groups
2004	Guide to the methods of technology appraisal (2nd ed.) Guide to the technology appraisal process (2nd ed.) Technology appraisal process: guidance for appellants	A guide for manufacturers and sponsors A guide for healthcare professional groups A guide for NHS organisations A guide for patient/carer groups
2005	Social value judgements: Principles for the development of NICE guidance (1st ed.)	
2006	Guide to the single technology appraisal process (1st ed.)	
2007		Single Technology Appraisal Process: update
2008	Guide to the methods of technology appraisal (3rd ed.) Social value judgements: Principles for the development of NICE guidance (2nd ed.)	
2009	Guide to the single technology appraisal process (2nd ed.) Guide to the multiple technology appraisal process (3rd ed.)	Supplementary advice: appraising life-extending, end-of-life treatments
2010	Appeals process guide	
2011		Clarification on discounting
2013	Guide to the methods of technology appraisal (4th ed.)	
2014	Guide to the processes of technology appraisal (4th ed.) Guide to the technology appraisal and highly specialised technologies appeal process	
2016		Addendum A: Final amendments to the NICE technology appraisal processes and methods guides to support the proposed new Cancer Drugs Fund arrangements Rapid re-consideration of drugs currently funded through the Cancer Drugs Fund
2017		Fast track appraisal: addendum to the Guide to the processes of technology appraisal Cost comparison: addendum to the Guide to the processes of technology appraisal Procedure for varying the funding requirement to take account of net budget impact
2018	Guide to the processes of technology appraisal (4th ed.) – 2018 update	

## Footnotes

1 The current review of NICE’s process and methods for health technology evaluation was initiated in July 2019, with changes due to be implemented in January 2022. It has been led by NICE with oversight from a steering group (consisting of both internal and external members) and with input from several external stakeholder groups. Topics of focus for the methods review have included the ‘modifiers’ considered in decision-making (that is, value-based criteria for exceeding the standard cost-effectiveness threshold such as disease severity, health inequality, rarity and innovation), treatment of uncertainty, types of evidence, health-related quality of life, technology-specific issues (such as the consideration of histology-independent cancer treatments) and discounting. Topics of focus for the process review include overall simplification of the health technology evaluation process, the process for consideration of highly specialised technologies and the negotiation and operation of managed and commercial access agreements [25]. Early outputs from the review suggest that several of the trends previously highlighted by the previous study [2] are likely to continue in the future. In particular, the ‘case for change’ emerging from the methods review includes increased reliance on formal decision-rules to set out how the needs of different groups should be prioritised and an expansion of the circumstances in which “a greater degree of uncertainty and risk should be accepted” in recommending a technology [26]. Outputs from the process review indicate a shift towards greater process alignment, increased flexibility and faster decision-making [27].

2 The three “moral principles” included in the final version appear to be loosely based on Beauchamp and Childress’s four principles of medical ethics. However, while SVJ provided further specification of the ‘justice’ principle through its explicit presentation of the AfR and EOC frameworks, this is not the case in Principles.

3 The EOL criteria currently apply to technologies which offer at least a three-month extension to life for a patient population with a life expectancy of two years or less. Application of the criteria allow QALYs gained at the end of life to be weighted by a factor of up to 1.7, effectively increasing the cost-effectiveness threshold to around £50,000/QALY [20].

4 This decision-rule is treated by NICE as a technical adjustment, presented through sensitivity analysis, rather than a normative judgement [22]. However, there is strong evidence to suggest that its introduction was motivated by normative concerns [2, 37, 38] and it nevertheless has the effect of prioritising the needs of a particular group based on considerations usually deemed to be morally relevant. i.e. life expectancy and disease severity.

5 The CDF is a source of ring-fenced funding for NHS cancer patients in England. It was established in 2011 and has been administered in part by NICE since 2016.

6 A 2017 review of NICE’s work identified 13 separate process and methods guides from across six programmes, excluding those focused purely on the appeals process or on public health and social care [4]. This figure also excludes five more recent addenda that were identified by the previous study and which must be read in conjunction with the main guides in order to obtain a full account [2]. The current methods guide for the core appraisal programme is 94 pages [22], while the process guide is 104 pages [28]. For the diagnostics assessment programme, the combined process and methods guide extends to 130 pages [29], while the combined guidelines manual is 240 pages long [30]. The ongoing process and methods review is intended to consolidate some of these documents; however, it seems likely that the combined guidance will continue to extend to several hundred pages. [25–27].

7 For example, the normative view that productivity costs should be excluded from the calculation of a technology’s cost-effectiveness is currently made in a single sentence, 45-pages into a 95-page methods guide, in highly technical section focused primarily on evidence synthesis [22]. Similarly, the normative implications of the “non-reference-case discount rate” applied in certain circumstances is not made explicit in the guide [22].

8 Certainly, SVJ’s claim that NICE does not prioritise drugs for rare diseases is factually inaccurate given the approach taken by the HST programme [21, 36].

9 NICE’s apparent shift away from openly articulating its reasoning about the substantive basis for decision-making could also be interpreted as being driven by epistemic factors; for example, in response to a perceived rejection of expertise and increased scrutiny of the role of science in policy-making (particularly in light of the current Covid-19 pandemic). This interesting line of thought is worthy of further investigation but goes beyond the scope of this paper and has therefore been left for others to explore.
